# Framing Health Care Instruction: An Information Literacy Handbook for the Health Sciences

**DOI:** 10.29173/jchla29604

**Published:** 2022-04-01

**Authors:** Cari Merkley

**Affiliations:** University Library Mount Royal University, Calgary, AB, Canada

Young LM & Hinton EG. **Framing Health Care Instruction: An Information Literacy Handbook for the Health Sciences**. Lanham: Rowman & Littlefield; 2019. Softcover: 154 pages. 9781538118931. Price: $63 USD. Available from: https://rowman.com/ISBN/9781538118948/Framing-Health-Care-Instruction-An-Information-Literacy-Handbook-for-the-Health-Sciences.

Many academic librarians will clearly remember the turmoil and anxiety that followed the Association of College and Research Libraries’ (ACRL) decision to replace its existing information literacy competency standards in 2015 with a *Framework for Information* *Literacy for Higher Education*. Checklist style standards that many institutions had used to organize their program of information literacy instruction were replaced with inherently nebulous threshold concepts that many were uncertain how to appropriately address and assess in their teaching. Several publications have since sought to provide practical examples of how the framework can be incorporated into information literacy instruction, including the ACRL’s own *Framing Information Literacy* six book series. Published in 2018 and edited by M.K. Oberlies and J. Mattson, each volume outlined a single frame and provided examples of appropriately tailored lesson plans from various disciplines. Similar in approach, *Framing Health Care Instruction: An Information Literacy Handbook for the Health Sciences* is a welcome addition to this body of work, providing a brief but focused discussion of the framework in a health sciences context along with practical advice on how to address it with students.

The book is part of the Medical Libraries Association series published through Rowman and Littlefield. Editors E.G. Hinton and L.M. Young are academic librarians based in the U.S. with experience working with students in the health sciences. It begins with examples of how existing statements in health science education accreditation standards can be mapped to the ACRL framework. This particular section will be of less value to Canadian readers because the focus is on U.S. based accreditation organizations, but the connections drawn might be illustrative to others doing similar work. This is a helpful reminder that leveraging accreditation or competency to practice standards in conversations around information literacy with faculty can broaden their understanding of what falls under information literacy and increase buy-in for instructional initiatives.

Subsequent chapters are organized around each of the six frames/threshold concepts, beginning with a short introduction and a helpful summary infographic of the frame by the book’s editors. This is followed by several examples of instruction sessions addressing that frame submitted by health librarians from different academic institutions across the U.S. and Canada. Each case study notes the frames addressed by the session(s), target audience, setting, learning outcomes, assessment strategies, and description of the session/activities. The chapter on the frame “Research as Inquiry” is the exception to this pattern - instead of the editors providing a brief introduction, authors Yost and Sade begin the chapter with an expanded case study of how the frame was addressed at Washington College in a first-year seminar on health and technology through faculty and librarian collaboration.

As a librarian working with undergraduate nursing and midwifery students, this book provided me with many ideas for changing up my own teaching. Many of the topics covered by the case studies will be very familiar - PICO, building search strategies, etc., but you will also find lessons on preparing for publication, predatory publishing, information privilege, and interprofessional education opportunities. It was the glimpse inside others’ classrooms via the provided case studies that were of most interest and use. The short introduction and particularly the summary infographic for each frame did serve as a helpful review. Those of us working with undergraduate health science students are particularly well served by the selected lesson plans, although those working with graduate students will also find examples in a variety of professional disciplines. As many of us have been moving between in person and online instruction over the course of the COVID-19 pandemic, it is worth noting that several of the case studies relate to online instruction or instruction that could be delivered in either environment.

There are a few issues to note. Since many of the case studies support multiple frames, the chapters do not truly stand alone, and the case studies can occasionally seem repetitive. The book’s brevity, which overall is a strength, also means that readers may be left wanting additional details on how instruction was delivered for some of the case studies. The utility of this book for those working with qualified health professionals rather than students may also be somewhat limited, as few case studies for this specific audience are provided.

This book will be valuable to experienced academic health librarians looking for ideas to refresh or enhance their instruction and for those new to teaching information literacy who are looking for a place to start. Those institutions who already own the *Framing Information Literacy* series will still find this a worthwhile purchase because of its disciplinary focus.

